# Coverage, use and maintenance of bed nets and related influence factors in Kachin Special Region II, northeastern Myanmar

**DOI:** 10.1186/s12936-015-0727-y

**Published:** 2015-05-21

**Authors:** Hui Liu, Jian-wei Xu, Xiang-rui Guo, Joshua Havumaki, Ying-xue Lin, Guo-cui Yu, Dai-li Zhou

**Affiliations:** Yunnan Institute of Parasitic Diseases; Yunnan Provincial Centre of Malaria Research, Yunnan Provincial Collaborative Innovation Centre for Public Health and Disease Prevention and Control, Yunnan Provincial Key Laboratory of Vector-borne Diseases Control and Research, Puer, 665000 China; Yangjiang County Centre for Disease Control and Prevention, Yangjiang, 679300 China; Foundation for Innovative New Diagnostics, 1216 Cointrin, Geneva, Switzerland

**Keywords:** Malaria, Bed net, Insecticide-treated nets (ITNs), War-torn population, Kachin Special Region II, Myanmar

## Abstract

**Background:**

Myanmar is one of the 31 highest burden malaria countries worldwide. Scaling up the appropriate use of insecticide-treated nets (ITNs) is a national policy for malaria prevention and control. However, the data on use, influencing factors and maintenance of bed nets is still lack among the population in Kachin Special Region II (KR2), Northeastern Myanmar.

**Methods:**

The study combined a quantitative household questionnaire survey and qualitative direct observation of households. A Chi-squared test was used to compare the percentages of ownership, coverage, and rates of use of bed nets. Additionally, multivariate logistic regression analysis (MVLRA) was used to analyse factors that influence the use of bed nets. Finally, covariance compared the mean calibrated hole indexes (MCHI) across potential influence variables.

**Results:**

The bed net to person ratio was 1:1.96 (i.e., more than one net for every two people). The long-lasting insecticidal net (LLIN) to person ratio was 1: 2.52. Also, the percentage of households that owned at least one bed net was 99.7 % (666/688). Some 3262 (97.3 %) residents slept under bed nets the prior night, 2551 (76.1 %) of which slept under ITNs/LLINs the prior night (SUITNPN). The poorest families, those with thatched roofing, those who use agriculture as their main source of family income, household heads who knew that mosquitoes transmit malaria and those who used bed nets to prevent malaria, were significantly more likely to be in the SUITNPN group. However, residents in lowlands, and foothills were significantly less likely to be SUITNPNs. Finally, head of household attitude towards fixing bed nets influenced MCHI (F = 8.09, *P* = 0.0046).

**Conclusions:**

The coverage and usage rates of bed nets were high, especially among children, and pregnant women. Family wealth index, geographical zones, household roofing, source of family income, household head’s knowledge of malaria transmission and of using bed nets as tools for malaria prevention are all independent factors which influence use of ITNs/LLINs in KR2. Maintaining high coverage, and use rate of bed nets should be a priority for the war-torn population of KR2 to ensure equity and human rights.

## Background

In the last decade, there has been encouraging progress in malaria control and prevention. Factors such as economic development, urbanization, and largely unprecedented financial support have all contributed to the success in the fight against malaria [1]. However, malaria is still a major global public health problem. In 2010, the worldwide estimated number of new cases of malaria was 219 million. This resulted in 660,000 deaths. In Southeast Asia (SEA) alone, 32 million new cases, and 43,000 deaths are estimated for 2010 [2]. The Greater Mekong Subregion (GMS) is a very high-risk region for malaria. Around 70 % of the total population is at risk of contracting malaria and 26 % are in a high-risk area (i.e., reported incidence of more than one case per 1000 people per year) [3, 4]. Myanmar is one of the 31 highest burden malaria countries worldwide. Over the past three decades, the number of malaria cases and malaria-induced mortality in SEA has been highest in Myanmar [3, 5]. Importantly, malaria in Myanmar affects transmission in neighbouring countries too. This seriously impedes malaria elimination efforts in these countries [6]. The WHO Global Malaria Programme has recommended full coverage of long-lasting insecticidal nets (LLINs) in areas targeted for malaria prevention [7]. Also, scaling up appropriate use of insecticide-treated nets (ITNs) is a national policy in Myanmar aimed at preventing and controlling malaria. Implementation strategies include free delivery of LLINs and free treatment of mosquito nets already in use before the start of the peak transmission season. These interventions are aimed at ensuring that people living in high-risk malaria areas can protect themselves. The goal is that at least 80 % of people in moderate and high-risk areas are protected by ITNs/LLINs by 2015 [8]. Kachin State is a bordering region that is heavily forested area and has a high concentration of poor ethnic minorities. The malaria burden is particularly high and malaria outbreaks occur frequently. In 2005, the mortality rate of malaria was as high at 7.8 deaths per 1000 people [3, 5, 9]. However, coverage, use of bed nets, and other factors that influence malaria prevention and control have not been well studied among the war-torn population of Kachin Special Region II (KR2). The objective of this study was to research the coverage of both treated (ITNs and LLINs) and untreated bed nets, and also to determine which factors influence bed net use and maintenance. This field study was conducted from June to September 2013 during the high-risk season for contracting malaria.

## Methods

### Study site

**Fig. 1:**
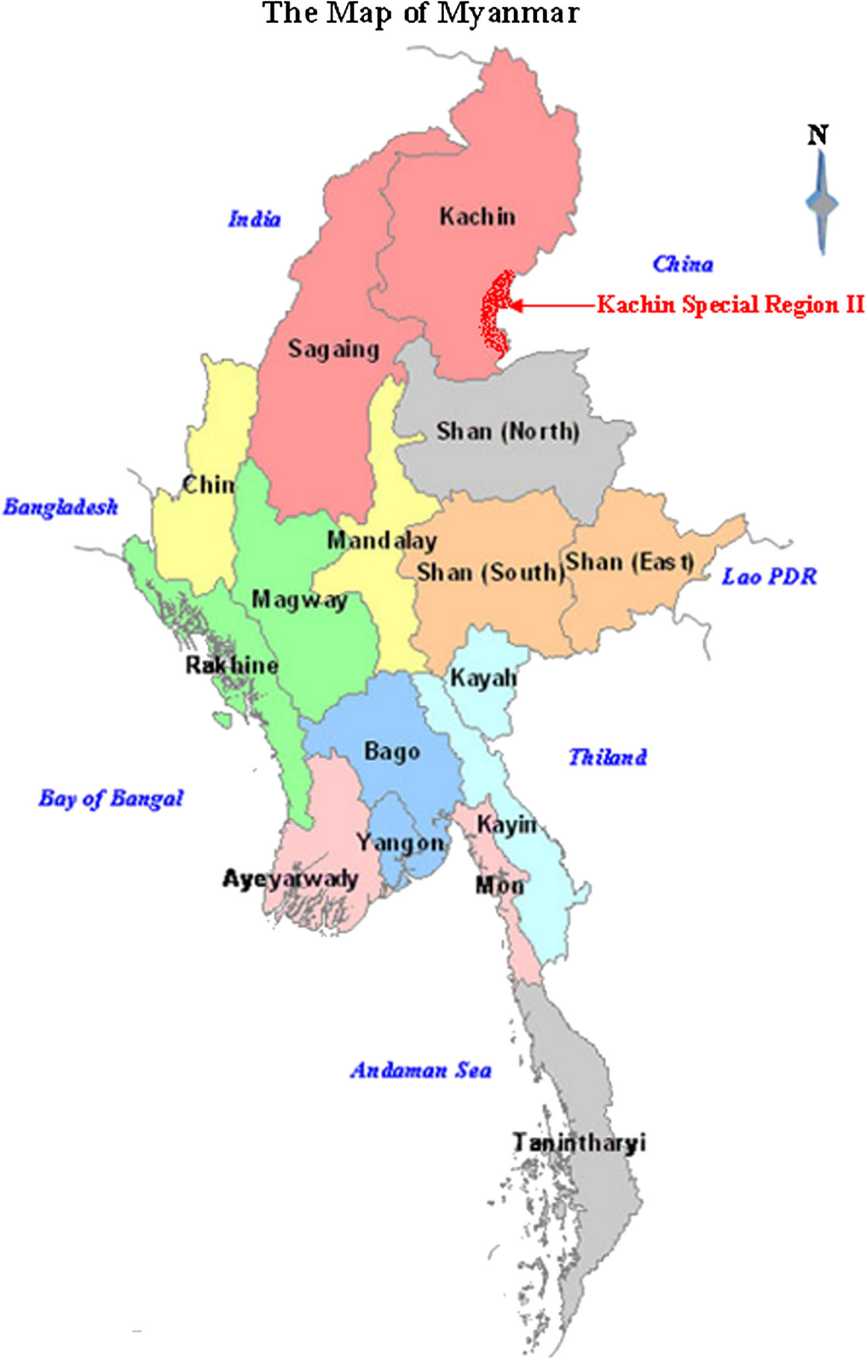
The study site: Kachin Special Region II, Myanmar

This study was conducted in KR2 along the China-Myanmar border in a hilly and forested area with a tropical rainforest climate (Fig. 1). The total population is about 60,000 and most of them are Kachin Ethnic Minority (known as Jinghpaw in China). In KR2, hot weather, pluviosity, and a dense forest facilitate the breeding and development of anopheline mosquitoes. Malaria vectors are complex, and the primary vectors are *Anopheles dirus* and *Anopheles minimus*. Malaria transmission occurs the whole year, but the peak occurs during the rainy season from May to November each year. During that time, *Plasmodium falciparum*, *Plasmodium vivax, Plasmodium malariae,* and *Plasmodium ovale* are all prevalent [8]. In the region, malaria control programmes are generally provided by international non-governmental organizations (INGOs).

### Household survey

The study combined a quantitative household questionnaire survey and qualitative direct observation. The questionnaires were developed in Chinese. The surveys were conducted by researchers from Yingjiang County Centere for Disease Control and Prevention of China who understand both Kachin/Jinghpaw language and Chinese. The interviews were conducted in the Kachin language and then the questionnaire was filled in Chinese. The study design was a two-stage cluster survey: stage 1 for selection of villages and stage 2 for households. Households were the sampling units. The list of village names was obtained through the Department of Health in KR2. Thirty-three out of 296 villages were selected by a simple computer randomization. The village Census was obtained from each village head. Next, surveys were conducted in 20 households in each selected village that were selected by simple computer randomization. A sample size of 660 households was required for the 5 % precision around a 25 % point estimate of the proportion of community residents sleeping under intact ITNs/LLINs the prior night, 95 % confidence limits and assumed 90 % of response rate. A household was defined as all those ‘eating from the same pot’. The interviewers first introduced the purpose of the project, the topic, and type of questions. An oral, informed consent was obtained, and finally the questionnaire was administered to each head of household. After permission was obtained, all bed nets in each household were inspected to determine how much they had been used, and how many had holes.

In the survey, holes were first categorized as either head-sized holes, hand-sized holes, or finger-sized holes, and then counted. The ratio between the observed numbers of holes of different sizes relative to the number of finger-sized holes was used to calculate an overall hole index. This process was assumed to be a reasonable proxy for the relative rate at which different classes of holes accumulate in domestic use. Among the inspected bed nets, there were three times as many finger-sized holes as hand-sized holes, and 12.9 times as many finger-sized holes as head-sized holes. The hole index (HI) for each net was calculated as: HI = number of finger-sized holes + 3 × number of hand-sized holes + 12.9 × number of head-sized holes [10].

### Concept definitions

Nets were classified in three ways; untreated nets, expired ITNs/LLINs, and ITNs/ LLINs that had not expired. An untreated net was never treated with insecticide. Expired ITNs had been treated with insecticide 12 or more months prior to the survey. An ITN was treated less than 12 months prior to the survey. There is a gradual loss of insecticide over time in LLINs, reducing their protective effect. LLINs therefore expire three or more years after their production. However, the dates of LLIN production could not be identified in the survey, so expired LLINs were defined as those that had been owned for more than three years. To decrease recall bias, bed net use was determined by asking which individuals slept under a net during the night prior to the survey. Family wealth index (FWI) was determined by household characteristics, such as housings, walls, and roofs, and assets, such as bicycle, and then classified into five groups (Table 1) [11–13]. The number of mice indoors was determined by asking the respondents the question: “Are there many mice in your house?” The four selectable answers were (1) never see, (2) see few, (3) see commonly, and (4) see many; (1) and (2) were combined into the category ‘few’, (3), and (4) into the category ‘common’ for analysis.

**Table 1:** Principal components for construction of family wealth index (FWI)

Family wealth index	Housing characteristics	Transportation tools	Family belongings
1 Most poor	Bamboo walls and thatch roofs	None	None or chickens
2	Wood walls and thatch roofs	Bicycles	Pigs or goats
3	Wood walls and terracotta roofs	Motorcycles	Cattle or horses
4	Brick walls and terracotta roofs	Tractors,	TV sets or refrigerators
5 Least poor	Steel and concrete	Cars	Elephants

### Data analysis

Double data entry and cleaning of quantitative data was done in Epidata 3.1. The dataset was then analysed in EpiInfo 2000. Household ownership, coverage, and use of untreated nets, expired ITNs/LLINs, and ITNs/LLINs were analysed. A Chi-squared test was use to compare the percentage of those that owned bed nets and use of bed nets across different demographic groups. Sleeping under ITNs or LLINs the prior night (SUITNPN) was the outcome variable and a multivariate logistic model was used to assess the association of the SUITNPN and the exposure variables. Covariance was also used to compare the mean calibrated HI across types of household roofs, numbers of chickens, and ducks, amount of mice indoors and the head of household attitude towards fixing bed nets [14].

### Ethical approval

According to the Helsinki Declaration, ethical approval for the study was granted by the Ethics Committee of Yunnan Institute of Parasitic Diseases (YIPD) of China. The research protocol was approved by the Health Department of Kachin Special Region II (HDKR2). The Ethics Committee of YIPD and HDKR2 approved a verbal consent procedure as sufficient because the study was interview-based, and did not include any human specimens. The purpose and procedures of the study were explained and disclosed to all participants before obtaining informed consent. Individuals could choose whether or not to participate in the study, and could refuse to respond to any question. The participants were asked for oral consent at the start of the survey and agreement to report their bed net data, and also notified that they could skip questions or end the interview at any time. Their consent was assumed if they did not refuse to answer questions. No one was coerced to participate in the study and if individuals wanted to withdraw, they were allowed to do so without any issue.

## Results

### Characteristics of respondents

Some 668 households with a total 3351 individuals participated in the study. Six-hundred and ten (91.3 %) of the households were from the Kachin Ethnical Minority, and other ethnics included Chinese, Shan, and Lisu. Questionnaires were administered to 144 (21.6 %) male and 524 (78.4 %) female heads of households. The mean age of the 668 respondents was 41.1 (median 39.0, range 16–76) years old; 435 (65.1 %) heads of households were 35 years or older; 144 (21.6 %) of the respondents were illiterate, 354 (63 %) had one to six years of school education and 170 (25.4 %) had ≥ seven years; 614 (97.0 %) households were classified as the most poor (FWI = 1-2) (Table 2).

**Table 2:** Household ownership of bed nets in Kachin Special Region II, northeastern Myanmar

	No. households without bed nets (%, 95 % CI)	No. households with untreated bed nets (%, 95 % CI)	No. households with expired ITNs/LLINs (%, 95 % CI)	No. households with ITNs/LLINs (%, 95 % CI)
Sex of household head				
Male (*n* = 144)	1 (0.7, 0–3.8)	59 (41, 32.9–49.5)	36 (25, 18.2-32.9)	126 (87.5, 81–92.4)
Female (*n* = 524)	1 (0.2, 0–1.1)	185 (35.3, 31.2–39.6)	20 (3.8, 2.3–5.8)	477 (91, 88.3–93.3)
*x* ^2^ value	-	1.56	66.0	1.60
P-value	-	0.2110	<0.0001	0.2054
Age of household head (years)				
<35 (*n* = 233)	0 (0, 0–1.6)	89 (38.2, 31.9–44.8)	34 (14.6, 10.3–19.8)	208 (89.3, 84.6–92.9)
≥35 (*n* = 435)	2 (0.5, 0.06–1.7)	155 (35.6, 31.1–40.3)	22 (5.1, 3.2–7.6)	395 (90.8, 87.7–93.3)
*x* ^2^ value	-	0.43	17.96	0.41
P-value	-	0.5117	0.00002	0.5237
Ethnics				
Kachin (*n* = 610)	2 (0.3, 0.04–1.2)	303 (49.7, 45.6–53.7)	54 (8.9, 6.7–11.4)	556 (91.1, 88.6–93.3)
Others^a^ (*n* = 58)	0 (0, 0–6.1)	24 (41.4, 28.6–55.1)	2 (3.4, 0.4–11.9)	47 (81.0, 68.6–90.1)
*x* ^2^ value	-	1.46	1.37	6.17
P-value	-	0.2273	0.2415	0.0130
School education years				
Illiterate (*n* = 144)	0 (0, 0–2.5)	52 (36.1, 28.3–44.5)	1 (0.7, 0–3.8)	126 (87.5, 81–92.4)
1–3y (*n* = 129)	1 (0.8, 0.02–4.2)	49 (38, 29.6–46.9)	2 (1.6, 0.2–5.5)	120 (93, 87.2–96.8)
4–6y (*n* = 225)	0 (0, 0–1.6)	75 (33.3, 27.2–39.9)	40 (17.8, 13–23.4)	216 (96, 92.5–98.2)
7–9y (*n* = 117)	1 (0.9, 0.02–4.7)	42 (35.9, 27.2–45.3)	13 (11.1, 16.1–18.3)	106 (90.6, 83.8–95.2)
≥10y (*n* = 53)	0 (0, 0–6.7)	26 (49.1, 35.1-63.2)	0 (0, 0–6.7)	35 (66, 51.7–78.5)
*x* ^2^ value	3.45	4.73	50.76	46.23
*P*-value	0.4849	0.3164	<0.0001	<0.0001
Family wealth index				
1 Most poor (*n* = 498)	1 (0.2, 0–1.1)	175 (35.1, 30.9–39.5)	28 (5.6, 3.8–8)	467 (93.8, 91.3–95.7)
2 (*n* = 116)	0 (0, 0–3.1)	51 (44, 34.8–53.5)	27 (23.3, 15.9–32)	113 (97.4, 92.6–99.5)
3 (*n* = 24)	1 (4.2, 0.1–21.1)	10 (41.7, 22.1–64.4)	1 (4.2, 0.1–21.1)	17 (70.8, 48.9–87.4)
4 (*n* = 10)	0 (0, 0–60.2)	8 (80, 44.4–97.4)	0 (0, 0–60.2)	6 (60, 26.2–87.8)
5 Least poor (*n* = 0)	0	0	0	0
*x* ^2^ value	12.19	11.11	38.77	39.06
*P*-value	0.0068	0.0111	<0.0001	<0.0001
Geographical zone				
Lowland (*n* = 226)	0 (0, 0–1.6)	102 (45.1, 38.5–51.9)	6 (2.7, 1.0–5.7)	202 (89.4, 84.6–93.1)
Foothill (*n* = 299)	0 (0, 0–1.2)	94 (31.4, 26.2–37.0)	11 (3.7, 1.9–6.5)	262 (87.6, 83.3–91.1)
Mid hill (*n* = 18)	1 (5.6, 0.1–27.3)	6 (33.3, 13.3–59.0)	8 (44.4, 21.5–69.2)	18 (100.0, 81.5–100.0)
Upper hill (*n* = 125)	1 (0.8, 0.02–4.4)	42 (33.6, 25.4–42.6)	31 (24.8, 17.5–33.3)	121 (96.8, 92.1–99.2)
*x* ^2^ value	19.29	11.10	92.01	10.59
*P*-value	0.0002	0.0112	<0.0001	0.0141
Total (*n* = 668)	2 (0.3, 0.04–1.1)	244 (36.5, 32.9–40.3)	56 (8.4, 6.4–10.7)	603 (90.2, 87.8–92.4)

^a^Others include Chinese, Shan, and Lisu; *95 %CI* 95 % confidence interval, *ITN* Insecticide-treated nets, *LLIN* long-lasting insecticidal nets

### Ownership and types of the bed nets

Ownership of bed nets was high in KR2, only two (0.3 %) households had no bed net and 666 (99.7 %) households owned at least one net. There was a total of 1714 nets among all households sampled; 244 (36.5 %) households owned at least one untreated net, 56 (8.4 %) owned at least one expired ITN or LLIN and 603 (90.2 %) owned at least one effective ITN or LLIN (Table 2). The ownership of effective ITNs/LLINs among the poorest families (FWI = 1-2) was significantly higher than ownership among wealthier ones (FWI = 3-5), 94.5 % (580/614) versus 67.6 % (23/34) (*x*^2^ = 31.82, P < 0.0001), respectively, with ownership of untreated bed nets among the former being slightly lower than the latter, 36.8 % (226/614) versus 52.9 % (18/34) (*x*^2^ = 3.57, *P* = 0.058), respectively. Overall, the bed net coverage was also high, with the net to person ratio at 1:1.96 (i.e., more than one net for every two people), and the LLIN to person ratio at 1: 2.52 (i.e., one LLIN for every two-and-a-half people).

According to villager-self report in the survey, 298 (17.3 %) nets were untreated, 85 (5.0 %) were expired ITNs, two (0.01 %) were expired LLINs and 1329 (77.5 %) were non-expired LLINs. Based on project records of LLIN distribution from Health Poverty Action, a UK-based INGO, a total of 35,297 LLINs were distributed from the sixth and tenth rounds of Global Fund to Fight AIDS, Tuberculosis, and Malaria (GFATM) for the China Malaria Programme during 2008 to 2013; the numbers for round 6 in 2008, 2009, 2010, and 2011 were 6197, 1396, 1328, and 1459, respectively; the numbers for round 10 in 2012 and 2013 were 18,145 and 6772, respectively. The records of LLIN distribution in each Village Health Station showed that the sixth and tenth rounds of GFATM delivered 103 (6.0 %) and 1239 (72.3 %), respectively nets to all the sampled households. The villagers themselves obtained 372 (21.7 %) commercial bed nets. This study did not collect the data on commercial net types because most bed nets were freely given LLINs from the GFATM. However, it was noticed during household visits that most of commercial nets were polyester-based and only a small portion of them were cotton. Some 13,019 (77.0 %) nets were owned for one to 12 months, 237 (13.8 %) for 13–24 months, 87 (5.1 %) for 25–36 months and 71 (4.1 %) for more than 36 months. One cotton bed net obtained by a villager had been owned for ten years and was still intact.

### Use of the bed nets

The rate of bed net use was high in KR2. Results from the survey revealed that 3262 (97.3 %) residents slept under bed nets the prior night (SUNPN), of which 2551 (76.1 %) SUITNPN. Overall, there was a significant difference (P < 0.0001) among four age groups. All 80 infants SUNPN, but 57 (71.1 %) of them SUITNPN and 23 (28.8 %) slept under untreated bed nets because their parents or caretakers were afraid that the ITN/LLIN insecticide was harmful; 1190 (98.4 %) children (one to 14 years) used bed nets; 313 (82.8 %) young children (one to four years) used ITNs or LLINs. Finally, adult males (≥15 years) had the lowest rate of LLIN use with 618 (70.5 %) SUITNPN. There was no significant difference between pregnant, non-pregnant and older female groups (*P* = 0.6807). Overall, of those that SUNPN, 40 (95.2 %) were pregnant females, 293 (93.0 %) were older females (≥50 years) - the lowest rate of bed net use, and the group with the highest rate was non-pregnant females with 717 (98.6 %) SUNPN (Table 3). Overall use of bed nets was not significantly different between residents in different geographical zones (*P* = 0.1253), numbers of SUNPN were 1101 (97.1 %) in lowland, 1453 (96.9 %) in foothills, 89 (98.9 %) in mid-hill and 619 (98.6 %) in upper hill areas, respectively. However, the residents in lowland, and foothills were significantly less likely to use ITNs/LLINs than in mid- and upper hills (P < 0.0001). Total number of residents SUITNPN was 840 (74.1 %) in lowland, 1111 (74.1 %) in foothills, 83 (92.2 %) in mid-hills and 517 (82.3 %) in upper hills. This distribution is a result of priority being given to mid and upper hill regions during the free mass LLIN distribution programme as they are the poorest and most vulnerable households (Table 3).

**Table 3:** Use of bed nets (sleeping under nets the prior night) among demographic groups in Kachin Special Region II, northeastern Myanmar

	Did not use	Use untreated bed nets	Use expired ITNs/LLINs	Use ITNs/LLINs
	(%, 95 % CI)	(%, 95 % CI)	(%, 95 % CI)	(%,95 % CI)
Infants (<1 year; *n* = 80)	0 (0, 0–4.5)	23 (28.8, 19.2–40)	0 (0, 0–4.5)	57 (71.3, 60–80.8)
Young children (1–4 years; *n* = 378)	9 (2.4, 1.1–4.5)	56 (14.8, 11.4–18.8)	0 (0, 0–1)	313 (82.8, 78.6–86.5)
Older children (5–14 years; *n* = 933)	12 (1.3, 0.7–2.2)	175 (18.8, 16.3–21.4)	1 (0.1, 0–0.6)	745 (79.8, 77.1–82.4)
Adult males (≥15 years; *n* = 876)	34 (3.9, 2.7–5.4)	224 (25.6, 22.7–28.6)	0 (0, 0–0.4)	618 (70.5, 67.4–73.6)
*x* ^2^ value	14.95	25.39	1.43	32.61
*P*-value	0.0019	0.0001	0.6984	<0.0001
Non-pregnant females (15–49 years; *n* = 727)	10 (1.4, 0.7–2.5)	174 (23.9, 20.9–27.2)	0 (0, 0–0.5)	543 (74.7, 71.4–77.8)
Pregnant females (*n* = 42)	2 (4.8, 0.6–16.2)	5 (11.9, 4–25.6)	2 (4.8, 0.6–16.2)	33 (78.6, 63.2–89.7)
Older females (≥50 years; n = 315)	22 (7, 4.4–10.4)	51 (16.2, 12.3–20.7)	0 (0, 0–1.2)	242 (76.8, 71.8–81.4)
*x* ^2^ value	23.14	10.15	49.71	0.77
*P*-value	0.0001	0.0062	<0.0001	0.6807
Lowland (*n* = 1134)	33 (2.9, 2.0–4.1)	260 (22.9, 20.5-25.5)	1 (0.1, 0–0.5)	840 (74.1, 71.4–76.6)
Foothill (*n* = 1499)	46 (3.1, 2.3–4.1)	341 (22.7, 20.6–25.0)	1 (0.1, 0–0.4)	1111 (74.1, 71.8–76.3)
Mid hill (*n* = 90)	1 (1.1, 0.03–6.0)	6 (6.7, 2.5–13.9)	0 (0, 0–4.0)	83 (92.2, 84.6–96.8)
Upper hill (*n* = 628)	9 (1.4, 0.7–2.7)	101 (16.1, 13.3–19.2)	1 (0.2, 0–0.9)	517 (82.3, 79.1–85.2)
*x* ^2^ value	5.73	25.45	0.51	32.07
*P*-value	0.1253	<0.0001	0.9168	<0.0001
Total (*n* = 3351)	89 (2.7, 2.1–2.3)	708 (21.1, 19.8–22.6)	3 (0.1, 0.02–0.3)	2551 (76.1, 74.6–77.6)

*95 % CI* 95 % confidence interval, *ITN* Insecticide-treated nets, *LLIN* long-lasting insecticidal nets

### Influence factors on use of the bed nets

The results of the multivariate logistic regression analysis (MVLRA) showed that FWI and residential location (geographic zone) were strongly associated with bed net use. The poorest families (FWI = 1-2) were significantly more likely to SUITNPN than wealthier families (FWI =3–5). The families in the lowlands and foothills were significantly less likely to use ITNs/LLINs because they had a higher FWI and more access to commercial bed nets than the families in mid- and upper hills before the mass free LLIN distribution. Therefore, they had a lower chance to receive LLINs during the distribution campaign. MVLRA identified that household roofs and family income sources were significantly associated with bed net use too. Individuals in households with thatched roofs and agriculture as the main source of family income source were significantly more likely to SUITNPN. Finally, heads of households who knew that mosquitoes transmit malaria and whether heads of households used bed nets to prevent malaria were identified as two independent factors for ITN and LLIN use (Table 4).

**Table 4:** Variables related to use of nets in Kachin Special Region II, northeastern Myanmar

	SUITNPN (%,95 % CI)	Univariate OR (95 % CI)	Adjusted OR (95 % CI)	*P* values
Sex				
Male (*n* = 720)	508 (70.6, 67.1–73.9)	0.68 (0.56–0.82)	0.98 (0.76–1.67)	0.8543
Female (*n* = 2620)	2043 (78.0,76.3–80.0)	1	1	
Age (years)				
<35 (*n* = 1165)	894 (76.7,74.2–79.1)	1.03 (0.89–1.22)	1.13 (0.99–1.72)	0.7915
≥35 (*n* = 2175)	1657 (76.2,74.3–78.0)	1	1	
School education years				
Illiterate and ≦3 years (*n* = 1366)	1013 (74.2,71.7–76.5)	0.82 (0.69–0.96)	0.96 (0.64–1.56)	0.5146
≥4 years (*n* = 1975)	1538 (77.9,76.0–80.0)	1	1	
Ethnics				
Kachin (*n* = 3050)	2338 (76.7,75.1–78.1)	1.22 (0.92–1.61)	1.12 (0.84–1.71)	0.1770
Others (*n* = 292)	213 (72.9,67.5–78.0)	1	1	
Family wealth index				
1-2 (*n* = 3070)	2445 (79.6,78.2–81.0)	6.05 (4.63-7.91)	4.67 (3.59–9.12)	<0.0001
3-5 (*n* = 270)	106 (39.3,33.4–45.4)	1	1	
Hill zone				
Lowland and foothill (*n* = 2625)	1951 (74.3,72.6–76.0)	0.56 (0.45–0.70)	0.63 (0.44–0.71)	<0.0001
Mid and upper hill (*n* = 716)	600 (83.9,80.9–86.4)	1	1	
House roof				
Thatch (*n* = 2820)	2202 (78.1,76.5–79.6)	1.77 (1.43–2.17)	1.57 (1.33–2.24)	<0.0001
Others (*n* = 522)	349 (66.9,62.6–70.9)	1	1	
Window and door screen				
Yes (*n* = 56)	46 (82.1,69.6–91.1)	1.45 (0.70–3.08)	1.25 (0.68–3.16)	0.3444
No (*n* = 3295)	2505 (75.8,74.3–77.2)	1	1	
Family income source				
Agriculture (*n* = 3092)	2395 (77.5,75.9–78.9)	1.96 (1.48–2.60)	1.66 (1.45–2.70)	<0.0001
Others (*n* = 245)	156 (63.7,57.3–69.7)	1	1	
Household heads knew mosquitoes transmitting malaria				
Yes (*n* = 3223)	2475 (76.8,75.3–78.2)	2.26 (1.55–3.30)	1.88 (1.45–3.47)	<0.0001
No (*n* = 128)	76 (59.4,50.3–68.0)	1	1	
Household heads knew bed nets against malaria infection				
Yes (*n* = 2534)	1806 (71.3,69.5–73.0)	0.24 (0.18–0.31)	0.58 (0.11–4.37)	0.4687
No (*n* = 816)	745 (91.3,89.2–93.1)	1	1	
Household heads took bed nets as tools for preventing malaria				
Yes (*n* = 3155)	2428 (78.0,75.4–78.4)	1.79 (1.30–2.47)	1.56 (1.22–2.67)	0.0003
No (*n* = 189)	123 (65.1,58.3–71.9)	1	1	

*95 % CI* 95 % confidence interval, *SUITNPN* sleeping under ITNs or LLINs the prior night, *OR* odds ratio

### Intactness of bed nets and related to factors

**Fig. 2:**
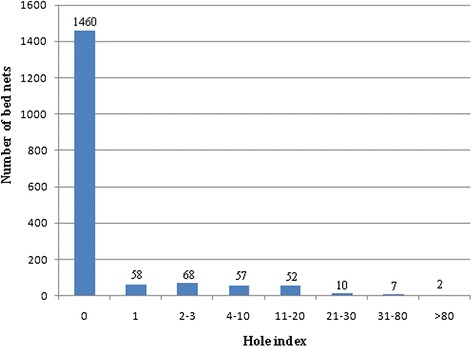
Number of bed nets by hole index

Among a total of 1714 bed nets, 1460 (85.2 %) were intact (HI = 0), and 254 (14.8 %) had holes. Only 71 (4.1 %) bed nets had a HI ≥ 10 (Fig. 2). The mean calibrated hole index (MCHI) of all bed nets was 1.33. The results of covariance revealed that roof type and numbers of chickens and ducks were not significantly associated with MCHI. However, the willingness of the head of household to repair bed nets was an independent influence factor for bed net intactness. The MCHI of bed nets was significantly lower when the head of household intended to repair bed nets than those without intent (F = 8.09, *P* = 0.0046). The number of mice indoors slightly influenced bed net intactness. The MCHI of bed nets was slightly higher in houses that mice could be seen ‘commonly’ than those where only ‘few’ mice were seen (F = 3.67, *P* = 0.0559) (Table 5).

**Table 5:** Influence factors on intactness of bed nets in Kachin Special Region II, northeastern Myanmar

	No. households (%,95 % CI^a^)	No. bed nets with				Mean calibrated hole index	Variance	F -value	*P* -value
		No holes	Finger-sized holes^aa^	Hand-sized holes^aa^	Head-sized holes^aa^				
House roof^a^									
Thatch	563 (84.3, 81.3–87.0)	1232	100	58	53	2.79	133.57		
Others	105 (15.7, 13.0–18.7)	228	19	11	13	1.53	13.60	1.23	0.2671
Number of chickens and ducks^a^									
<3	106 (15.9, 13.2–18.9)	234	17	12	12	3.48	68.51		
≥3	562 (84.1, 81.1–86.8)	1226	102	57	54	2.43	124.57	0.85	0.3562
Indoor abundance of mice^a^									
Few	209 (31.3, 27.8–35.0)	457	37	20	21	1.42	38.41		
common	459 (68.7, 65.0–72.2)	1003	82	49	45	3.13	149.86	3.67	0.0559
Willingness for mending bed nets (n = 548)									
Yes	479 (87.4, 84.3–90.1)	1046	85	48	47	1.30	119.39		
No	69 (12.6, 10.0–15.5)	150	12	8	7	5.38	158.18	8.09	0.0046

^a^Number of households = 668 unless otherwise indicated; ^aa^There was at least one indicated size hole a net that might have other smaller holes too; *95 % CI* 95 % confidence interval

## Discussion

In the Union of Myanmar, malaria is considered a leading cause of morbidity and mortality [8]. It is also the leading respondent-reported cause of death from community-based survey [15]. The goal of the new national strategic plan is to reduce malaria morbidity and mortality by at least 50 % against the baseline level of 2007 by 2015. Use of ITNs is widely recognized as a main effective intervention to prevent malaria. High use rates of ITNs are an important goal of many malaria programmes [16]. Another goal is that at least 80 % of the people in moderate- and high-risk villages among 180 selected townships should be using ITNs for protection against malaria. Ownership of at least one bed net per household is a main indicator used to assess outcomes. The results of this study reveal that ownership of bed nets is very high (99.7 %). A total of 1714 bed nets were reported amongst 668 households sampled; 77.5 % of bed nets were LLINs. The overall, bed net to person ratio was 1:1.96 while the LLIN to person ratio 1: 2.52 in the KR2 region. The high level of coverage is a result of the sixth and tenth rounds of GFATM for the China Malaria Programme, which distributed 35,297 free LLINs through Health Poverty Action (HPA) in KR2 in the past five years (2008–2013). Most (24,917) of the bed nets were delivered through the tenth round of GFATM during 2012–2013. When commercial sectors are responsible for bed net availability, the ownership is concentrated among the richest families [17]. Therefore, high bed net availability does not necessarily mean higher coverage [18]. Giving special attention to the poorest and the most vulnerable populations is essential to ensure universal access to LLINs. In order to ensure equity, the HPA GFATM team, in cooperation with village leaders, carried out a survey assessing both the household economic situation and bed net coverage before LLIN distribution. This ensured that the priority was given to the poorest and the most vulnerable households during the distribution campaign. The higher ownership of treated ITNs/LLINs among poorer families and the higher ownership of untreated bed nets among wealthier families show the effectiveness of the campaign. Free distribution of LLINs promoted the equity in LLIN ownership, and gave the war-torn population an essential human right.

Access to an ITN within the household is considered a much more accurate indicator of ITN use than simply owning an ITN [16]. People are at different levels of risk for complications from malaria. Pregnant women, particularly primagravida with malaria, have a high risk of malaria being associated with more serious illness and low birth weights [10, 19, 20]. The study shows a high rate of bed net use (97.3 %) among children (98.4 %) and females (98.6 %) in KR2. There was not a significant difference in use of bed net between adult females groups. However, it is important to note that pregnant females had higher use rate of ITNs or LLINs (78.6 %). This may be one of outcomes from the health behaviour and education interventions by the Chinese GFATM Malaria Programme. In the past five years, HPA delivered 390 sessions of community health education, with 29,759 attendees, to promote the use of bed nets in KR2. Side effects (smell, dizziness, headache, and itchiness) from LLIN insecticide were reported as a barrier for ITN utilization [21]. Only 57 (71.1 %) infants used LLINs because their parents or caretakers were afraid that the insecticide would be harmful. The side effect of insecticide is an issue that has received little attention from both academic and health authorities. Additionally, there have been incidents resulting from the use of LLIN insecticides during the implementation of malaria control programmes. For example, an outbreak of rash occurred in a school after LLIN distribution by a NGO in Mangshi City of Yunnan Province, China in 2009. More than 250 schoolchildren had itchy skin rash which resulted from allergic reaction to the insecticide in LLINs because the NGO did not teach the children to hang LLINs in ventilated places for 24 h before using them. Pyrethroid is usually used for treatment of bed nets and is relatively safe to humans. However strong smells and hypersensitivity of the eyes, skin, and respiratory system can occur; thus, safer insecticides should be developed for treatment of bed nets.

The MVLRA did not identify sex as an independent influence factor on use of ITNs/LLINs. The rate of use of ITNs/LLINs was the lowest (70.5 %) amongst adult males (≥15 years). Due to the war, most males are recruited into Kachin Independent Army. Therefore, they often go into the forest and back to their villages. They may carry malaria from the forest into their community. Further, it has been reported that males were more likely than females to have falciparum malaria infections (OR: 1.80; CI: 1.29, 2.54) in KR2 [22]. Bed net use within this community can prevent malaria transmission from the malaria-infected solders to civilians. Other measures, such as long-lasting insecticidal hammocks and topical and spatial repellents should be used by solders when they are in the forest [23–25].

Several authors have pointed out that household socio-economic status influences bed net access, and is the strongest determinant of net use. Wealthier families are much more likely to use nets [11, 16, 18, 26–28]. These data in KR2 are contrary to past studies. The poorest families were significantly more likely to SUITNPN than the wealthier families because of free distribution of LLINs in KR2. The free distribution of LLINs is also the result of lower bed net ownership among wealthier families (67.6 %). There is a so-called ‘diversion effect’ that untreated nets may divert mosquitoes from one person using an untreated net to an unprotected person in the same room. However, if the net is treated, there is evidence that the diversion effect does not occur because the insecticide offers some protection to the individual not using a bed net [10, 29]. The results showed that no residents used treated ITNs. Dipping existing nets and expired ITNs should be used in vector control measures for malaria prevention. The results of MVLRA showed that geographical zone, roof types, and family income sources were strongly associated with use of ITNs/LLINs. Families in the lowlands and foothills were significantly less likely to use ITNs and LLINs. The authors found that malaria prevalence is usually higher in lowland and foothill regions than mid- and upper hill areas. The MVLRA also identified that heads of households who knew that mosquitoes transmit malaria and that bed nets are the tool to prevent malaria were two independent factors for use of ITNs/LLINs. More than other vector control methods, ITN programmes largely depend on the acceptance and active involvement of communities. Involvement of communities in promoting use of ITNs also depends on their knowledge, perception, attitude, and behaviour towards nets [30, 31]. Knowledge, information and distribution of free bed nets are established as the first step towards encouraging ITN use [32]. As suggested by villagers and Health Department of KR2, interventions targeting health behaviour, and health education should be further strengthened in KR2.

The results showed that 1556 (90.8 %) of bed nets were owned for only two years, 158 (9.2 %) were owned for three years or more, and the mean calibrated HI was 1.33. The attitudes of heads of households towards fixing bed nets was an independent influence factor for net condition (F = 8.09, *P* = 0.0046). The amount of mice inside the household is significantly associated with bed net condition (F = 3.67, *P* = 0.0559). Not only is there a gradual loss of insecticide over time, but bed nets accumulate holes as a result of wear and tear. Both holes and loss of insecticide will reduce the protective effects of ITNs [10, 33]. When considering strategies to encourage behavioural changes, special attention should be given to education and what should be communicated [18]. Maintenance of bed net should be a behaviour change that is mentioned. During the interviews, respondents expressed concerns that the free LLINs were not strong enough. LLINs are easily torn and burnt by sparks from fire. They suggested that stronger bed nets should be distributed. Stronger fibres may ultimately be a more cost-effective option for the production and use of LLINs.

In spite of a 99.7 % ownership rate and 97.3 % (3262/3351) use rate of bed nets, with a 76.1 % (2551/3351) use rate of ITNs or LLINs, a malaria outbreak with 1093 confirmed malaria cases occurred in the lowland and foothill areas of KR2 during June and July 2013. The outbreak was finally controlled in August 2013 by using indoor residual spraying (IRS) of insecticides combined with standard anti-malarial treatment. The reasons of the outbreak in lowland and foothill areas may include (1) higher transmission intensity [34]; (2) higher population density and mobility; (3) lower proportion of ITN LLIN use, which was found to be 74.1 % (1951/2633) in lowlands and foothills versus 83.6 % (600/718) in mid and upper hills (Tables 3 and 5); and, (4) the principal vector, *An. minimus,* was found to bite people between nightfall and the time of people going to bed (about 22:00) in spite of its biting peak being from 23.00 to 01:00 [35], just as the respondents mentioned during interviews: “Bed nets cannot protect us from mosquito bites before we go to bed. Mosquitoes may bite the children when they play outdoors or are watching television. The feet, especially of children, may protrude out of the nets during sleeping”.

In 2014, the lesson from the previous outbreak was considered, and an IRS was carried out in late May 2014 when malaria incidence started to increase. This successfully prevented another outbreak. No significant benefit of combining LLIN and IRS compared with a background of LLIN coverage was documented in areas with low malaria vector density [36]. However, the combination of LLIN and IRS is effective to control potential outbreaks even when there is high LLIN coverage, and might be useful in settings of high malaria transmission. To ensure the success of vector control efforts, additional innovative strategies should be investigated [37, 38].

Study limitations include if demographic groups are not similar, bias might be introduced [39]. More females (524/668 = 78.4 %) were interviewed than males due to the fact that male heads of households are recruited into Kachin Independent Army and away from home during war time. This may cause selection bias and information bias. However, female heads of households should know about ownership and use of bed nets better than their husbands, meanwhile, the analysis adjusting for sex and age has been used to reduce the potential selection and information bias. Another limitation is that data on the number and/or type of net repaired, use of bed nets amongst children under five associated with increased MCHI have not been collected and assessed in this survey. Further survey on the issue may be valuable.

On the other hand, The National Malaria Control Programme (NMCP) of Myanmar hardly covers the KR2 because of the conflict between Kachin Independent Army and National Defense Army, therefore, 78.3 % (1342/1714) of the bed nets were from the sixth and tenth rounds of GFATM for China Malaria Programme. The GFATM stopped support China Malaria Programme in January 2014, and will not give further support to China. The existing LLINs will expire and they may be out of intactness within one year. Myanmar, China, and international societies should consider how to maintain the high coverage and use rate of bed nets, and other malaria control activities in KR2.

## Conclusions

Despite the fact that NMCP of Myanmar hardly covers KR2, the coverage, and use rates of bed nets was high, especially among children, and pregnant women. The free distribution of LLINs from the sixth and tenth rounds of GFATM for the China Malaria Programme ensured that the poorest and most vulnerable populations obtained universal access to LLINs in KR2. FWI, geographical zones, household roofs, family income sources, and head of household’s knowledge of malaria transmission and prevention through the use of bed nets are independent factors which influence use of ITNs/LLINs. Finally, attitudes of heads of households towards fixing bed nets influenced the net intactness. Therefore, special attention to maintaining the high rate of bed coverage and use should be given to the war-torn population to ensure equity and their human rights.
